# High risk, low rest: a new framework for monitoring sleep vulnerability in emergency medicine

**DOI:** 10.3389/fpubh.2025.1679296

**Published:** 2025-10-31

**Authors:** Laura Schmidt, Marion Trousselard, Clément Perez, Eve Reynaud, Bérénice Valero, Sophie Schlatter, Karim Tazarourte, Marion Douplat, Stéphanie Mazza

**Affiliations:** ^1^Forgetting Team, Centre de Recherche en Neurosciences de Lyon CRNL U1028 UMR5292, INSERM, CNRS, Université Claude Bernard Lyon 1, Bron, France; ^2^Research on Healthcare Performance (RESHAPE), INSERM U1290, Université Claude Bernard Lyon 1, Lyon, France; ^3^Institut de Recherche Biomédicale des Armées, Brétigny-sur-Orge, France; ^4^Inserm, INSPIIRE, University of Lorraine, Nancy, France; ^5^École de Psychologues Praticiens, Catholic Institute of Paris EA Religion, Culture et Société, Paris, France; ^6^Emergency Department, Hospices Civils de Lyon, Lyon, France; ^7^Human Science Department, Centre Léon Bérard, Lyon, France; ^8^Hospices Civils de Lyon, University Claude Bernard Lyon 1, Healthcare Simulation Center (CLESS), SIMULYON, Lyon, France

**Keywords:** shift work, sleep regularity, sleep vulnerability, fatigue risk assessment, actigraphy, healthcare, occupational health

## Abstract

**Background:**

Shift work in emergency care settings disrupts circadian rhythms and sleep, increasing health risks and performance. A key aspect of addressing these challenges lies in predicting the burden of shift work to develop safer schedules. This study introduces the Shift Load Index (SLI) as an advanced and sensitive metric for quantifying recovery constraints and examined its association with objective sleep outcomes in emergency healthcare professionals.

**Methods:**

A two-phase observational field study was conducted with 72 nurses and physicians from two French emergency departments. In the theoretical validation phase, 140 work shifts were analyzed using the SLI and compared to validated FAID Quantum fatigue scores. In the behavioral validation phase, weekly actigraphy data from 35 participants were analysed to assess time in bed, total sleep time, and Sleep Regularity Index (SRI). We employed generalized linear mixed-effects models to assess the association of SLI with sleep outcomes.

**Results:**

SLI scores significantly predicted FAID Quantum scores (all *p* < 0.001). Emergency healthcare professionals obtained on average 6h 09 min of sleep for 8h09min in bed, with irregular sleep patterns (mean SRI = 52%). Higher SLI scores were associated with reduced time in bed (*β* = −33.19, *p* < 0.001), shorter sleep (*β* = −18.30, *p* < 0.001), and lower SRI (*β* = −1.06, *p* < 0.001). SRI and total sleep time, as independent factors, together explained 48% of SLI variance (including random effects, 18% by fixed effects only).

**Discussion:**

Higher shift load is associated with both reduced sleep quantity and regularity. The SLI provides a useful tool to assess recovery burden, with potential applications in optimizing shift schedules and informing fatigue risk management strategies for emergency healthcare professionals.

## 1 Introduction

Over 15% of the active French workforce engage in night shifts, with the healthcare sector heavily relying on these workers (1). The demanding nature of shift work schedules, coupled with insufficient individual coping mechanisms, often leads to significant sleep disruption and circadian misalignment among emergency healthcare professionals (EHP); (2–4). Beyond the health concerns for the individuals themselves, insufficient sleep in EHPs has serious implications for professional performance and patient safety. There is well-documented evidence linking sleep disruption in EHPs to an elevated risk of medical errors, underscoring the critical need for effective fatigue management strategies (5–9). Despite existing regulations aimed at reducing the risks associated with shift work in healthcare (10, 11) the persistence of these issues points to the need for innovative solutions.

One limitation lies in the incomplete understanding of the dynamics between individual sleep patterns and the sleep opportunities provided by specific work schedules. Clarifying this relationship is crucial, as recovery management is a shared responsibility that hinges on the interplay between individual vulnerability factors (e.g., chronotype, sleep hygiene, and shift work tolerance) and institutional scheduling policies (9, 12, 64).

A key aspect of addressing these challenges lies in predicting the burden of shift work to develop safer schedules. This study applied an adapted Shift Load Index (SLI), originally derived from Gander’s fatigue risk matrix developed for the New Zealand healthcare sector (13, 14). Although the matrix proposed by Gander and colleagues is ecologically useful and practical compared to more complex algorithms, it has not yet been directly validated against objective sleep–wake data in field conditions. The SLI streamlines traditional biomathematical models by focusing specifically on recovery opportunities within shift schedules, excluding more complex factors like sleep cycles or traveling across time zones (15, 16). Grounded in Borbély’s two-process model for sleep regulation, the SLI captures the cumulative effects of homeostatic and circadian processes, known to effectively predict sleepiness without extensive data inputs (17).

Sleep duration and sleep regularity are particularly vulnerable to the erratic timing of rotating shift work. Actigraphy-based studies within the healthcare sector consistently reported that EHPs obtain less than the recommended 7 hours of sleep, particularly during night shifts (18–21). While some healthcare professionals achieve adequate recovery through extended rest periods, this often comes at the cost of increased sleep variability and delayed compensatory rest (8, 22).

A recent consensus of the National Sleep Foundation highlights the impact of sleep timing variability on health and performance (23) and mounting evidence suggests that sleep regularity may be a stronger predictor of long-term health outcomes, including mortality risk, than sleep duration alone (24). In this context, the Sleep Regularity Index (SRI) has emerged as a valuable metric for quantifying variability in sleep timing and duration (24, 25). By quantifying deviations from regular sleep–wake patterns, the SRI provides critical insights into the effect of rotating shifts on sleep stability. However, to our knowledge, it has not yet been specifically applied to capture vulnerability in shift workers.

Despite the relevance of both SLI and SRI, their relationship has yet to be systematically examined in the healthcare context. Given the potential impact of shift work on health and performance, further investigation is warranted. Indeed, in combination, the SLI and SRI present complementary perspective on recovery management among shift workers: the former indexing the structural demands of shift schedules, and the latter capturing their behavioral consequences on sleep stability.

Our investigation aims to clarify how shift-work schedules affect sleep patterns and evaluates individual sleep vulnerability. We introduce the SLI as an advanced metric for quantifying recovery constraints calibrated to European working-time regulations and validate it, both theoretically and behaviorally applied to the emergency department context. Theoretically, we assess the SLI’s capacity to reproduce predictions from the established FAID Quantum sleep-regulation model. Behaviorally, we test the hypothesis that the SLI covering a week schedule predicts two key objective sleep parameters over that week: sleep quantity (time in bed and total sleep time) and regularity (indexed by the SRI). By examining these dynamics, we aim to identify typical shift adaptation patterns within the French Emergency Department, contributing to safer scheduling practices and establishing individual adaptation margins.

## 2 Materials and methods

### 2.1 Study design and setting

The monocentric study REST (Recovery optimization in Emergency medicine addressing Stress adaptation Techniques) was approved by the Ethics Committee for the Protection of Individuals (2021-A03171-40) and was preregistered on ClinicalTrials.gov (Identifier: NCT05251246). REST was designed to measure the impact of a recovery intervention on EHP and data used for this naturalistic observational study was derived from the baseline measures. Data were collected for 2 weeks from staff working rotating shifts in two emergency departments of a university hospital of Lyon, France. The shift system requires nurses to rotate 12-h shifts (7:00 to 19:00 the day shift and 19:00 to 7:00 the night shift) and physicians to rotate between 10.5-h day shifts (8:00 to 18:30) and 14-h night shifts (18:30 to 8:30).

### 2.2 Selection of participants

In total, 72 volunteer EHPs were enrolled. Exclusion criteria were non-full-time staff (work time less than 80%) or staff that had worked less than a year in an emergency department. Participants were fully informed about the study and provided their voluntary, written informed consent to participate.

### 2.3 Data collection

Between February and November 2022, we collected both schedule data and objective sleep data, with shift rosters for SLI computation provided by emergency department and ambulance administration. The study was conducted in 2 phases: In the first phase (February–July 2022), we tested our index in a preliminary group of participants (early-stage subset) by comparing it to established risk measures (theoretical validation). In the second phase, following the completion of participant enrollment in November 2022 (REST protocol), we assessed the index’s relationship with objective sleep data collected at the pre-intervention stage for the full study sample (behavioral validation). Participants completed questionnaires at baseline (study inclusion) and again at the end of the sleep monitoring period. Demographic variables captured included sex, age, BMI, relationship status, presence of young children (≤ 5 years), shift-work experience, and commute time.

### 2.4 Chronotype

Diurnal preference was determined by the Morningness Evenigness Questionnaire MEQ (26). This validated questionnaire is comprised of 19 items. The total score ranges from 16 to 86 and defines the chronotype: scores ranging from 16 to 30 indicate extreme eveningness preference (E*); scores from 31 to 41 indicate evening preference (E); scores from 42 to 58 are categorized as neutral (N); scores from 59 to 69 indicate morning preference (M); and scores from 70 to 86 indicate extreme morning preference (M*).

### 2.5 Shift load index

The SLI is a validated predictive scoring system originally developed by Gander et al. (13, 14) to assess, based on planned work schedules, the imposed risk of limited recovery opportunities. For the present study, we adapted the SLI to the French shiftwork system and extended it by including a specific rating for the degree to which work hours conflict with typical social hours (evenings, weekends) recognizing that such conflicts may increase social pressure and further reduce sleep opportunities (27). These adaptations are detailed in a publicly available preprint (28). The SLI score generated for a work schedule of one week comprises 8 items, (Table 1); each with predefined cut-off scores to define 3 different risk levels: 0 (low risk), 1 (medium) or 2 (high). The higher the shift load, the greater the misalignment between work schedules and biological or social rhythms. Accordingly, higher risk levels represent less opportunity for recovery and restorative sleep. The total SLI score (ranging from 0 to 16) is obtained by summing the risk levels across all items. If possible, we also recommend including a ninth item in an extended version of the index to account for cumulative fatigue arising from the previous week. In the theoretical validation phase, the SLI score was computed based on two-week work schedules, allowing the use of the extended version rated on all nine items. In contrast, for the behavioral validation phase, the score was calculated from one-week work schedules to match the available behavioral data, and thus was rated on eight items only. Ratings were generated for each day using a dynamic sliding window approach, whereby each day’s score reflects recovery opportunities within the preceding seven-day period. This method enables a continuous and temporally sensitive assessment of recovery risk.

**Table 1:** Shift Load Index (SLI) items and risk levels for assessing a one-week work schedule.

Category	Items	Risk factor	0 – Low risk	1 – Moderate risk	2 – High risk
Work	1	Hours Worked	<40 h week	40-48 h week	>48 h week
	2	Long shifts	1 shift	2–3 shifts	>3 shifts
Rest	3	Longest Recovery Period	≥48 h	≤24 h	0
	4	Short Breaks	0	1	≥2
	5	Days fully rested	>1	1	0
Night	6	Night shifts	0	1–2	>2
	7	Biological sleep hours	<8 h lost	8 h lost	>8 h lost
Social life	8	Social hours	<8 h lost	8-13 h lost	>13 h lost

Long shifts >10 h. Longest recovery period assesses the consecutive time to disconnect and recover, similar to a typical weekend period. Short breaks <11 h. Biological sleep hours assumes one complete night of biological sleep amounts to 8 h between 23:00–7:00; hours lost refers to the cumulated hours of night time sleep duration during 1 week below 56 h (7 nights * 8 h), 8 h lost ≈ 1 complete night. Social hours are assumed to occur between 18:00 and 22:00 on weekday evenings and between 9:00 and 22:00 on weekends; hours lost refers to cumulated social hours below 46 h, 8 h lost ≈ two social evenings, 13 h lost ≈ a weekend day.

### 2.6 Theoretical validation

To validate the adapted SLI against an established biomathematical model commonly used in 24/7 industries, we included FAID Quantum scores for cross-measure comparison (29). The FAID Quantum scores are widely used biomathematical models in 24/7 industries. FAID Quantum estimates fatigue and alertness based on schedules derived from work / non-work hours and provides 2 primary metrics: (i) FAID-FATIGUE Score: Predicts on-duty physical fatigue based on the Fatigue Audit InterDyne algorithms; (0–100+; higher scores indicate higher predicted fatigue). Scores above 80 represent an established risk threshold validated by FAID Quantum; for comparison, a typical office week yields a score of approximately 41; (ii) FAID-ALERTNESS Score: Predicts on-duty sleepiness based on the Karolinska Sleepiness Scale (KSS) integrating the Three-Process Model of Alertness (1–9; higher scores indicate higher predicted sleepiness). The scores were obtained for work shifts only and not for days off.

### 2.7 Behavioral validation

Objective sleep data were obtained using an actigraphy device (Actiwatch 2; Philips - Respironics, Murrysville PA, USA). Participants wore the wrist device for 3 weeks and were asked to press a button to indicate bed time, defined as the moment when they attempted to fall asleep and again in the morning to indicate when they got out of bed (get up time). Sleep onset and Sleep Offset were defined the moment the subject fell asleep and woke up. Actigraphy monitors the activity level based on an accelerometer with data being collected at a frequency of 32 Hz and segmented to 30-s epochs. Data was analyzed using the Philips Actiware software version 6.0.1. Sleep regularity (SRI), total sleep time (TST) and time in bed (TIB) were measured whenever the sleep–wake pattern of an EHP included seven consecutive days of recording; all sleep types were cumulated (nighttime sleep, daytime sleep, sleep obtained at work, and naps, regardless of timing or setting). TST refers to the time asleep between the moment the EHP has fallen asleep (sleep onset) until the time they wake up (sleep offset), so any disrupted sleep (nocturnal awakenings) decreases the sleep quantity. TIB refers to the time the participant spends in bed from bedtime to get up time and represents the overall sleep opportunity.

SRI quantifies the regularity of sleep–wake patterns by comparing sleep/wake patterns over consecutive days (25, 30). It expresses the likelihood that any two time-points 24 h apart are in the same sleep or wake state. The SRI ranges from 0 (random pattern) to 100 (perfectly regularity) and is sensitive to naps, sleep fragmentation and non-consecutive data. Established cut-offs from larger cohorts exist for interpreting SRI scores as irregular, intermediate, or regular sleep patterns, typically based on the lowest and highest quintiles of the score distribution (30, 31). The SRI is computed with the following formula (Equation 1):


(1) $$-100+\frac{200}{M(N-1)}\sum_{j=1}^{M}\sum_{i=1}^{N-1}\delta (s_{i,j},s_{i+1,j})$$

where *M* is the number of daily epochs, N is the number of days, s_*i*,*j*_ = 0 for sleep and s_*i*,*j*_ = 1 for wake, and δ(s_*i*,*j*_, s_*i* + 1,*j*_) = 1 if s_*i*,*j*_ = s_*i* + 1,*j*_ (meaning the epochs separated by 24 h are in the same state) and 0 otherwise.

### 2.8 Data analysis

First, during the theoretical testing phase, the extended 9-item Shift Load Index (SLI) was compared to FAID Quantum scores, as FAID Quantum is also typically applied to two-week schedule data for comprehensive fatigue risk profiling. A generalized linear mixed-effects model (GLMM) was used, controlling for shift type and accounting for clustering by subject as a random effect (32–34).

Subsequently in the behavioral validation phase, the basic 8-item SLI was applied to objective sleep data over 1 week. Demographic group comparisons for sleep variables (SRI, TST, TIB) were conducted using *t*-tests or ANOVA, as appropriate. Sleep–wake patterns over time were visualized using locally estimated scatterplot smoothing (Loess). The predictability of sleep variables based on SLI scores was further tested with GLMMs, applying a sliding window approach with day-level data clustered by subject. Models were adjusted for demographic covariates, commute time, chronotype, and shift work experience. Model residuals were visually inspected using probability plots to assess normal distribution. A significance level of 0.05 was applied throughout.

Finally, to assess how sleep regularity and duration jointly predict shift-load scores, we performed an exploratory multivariable mixed-effects regression controlling for subject with daily SLI as the dependent variable and SRI and TST entered simultaneously as predictors.

## 3 Results

### 3.1 SLI – theoretical validation

**Figure 1:**
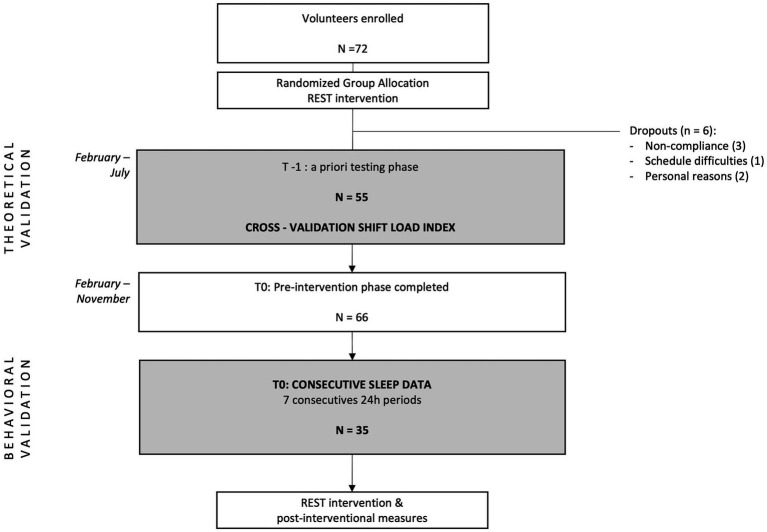
Flow chart of participant inclusion in the REST study up to baseline, showing screening, eligibility, and final sample sizes.

**Figure 2:**
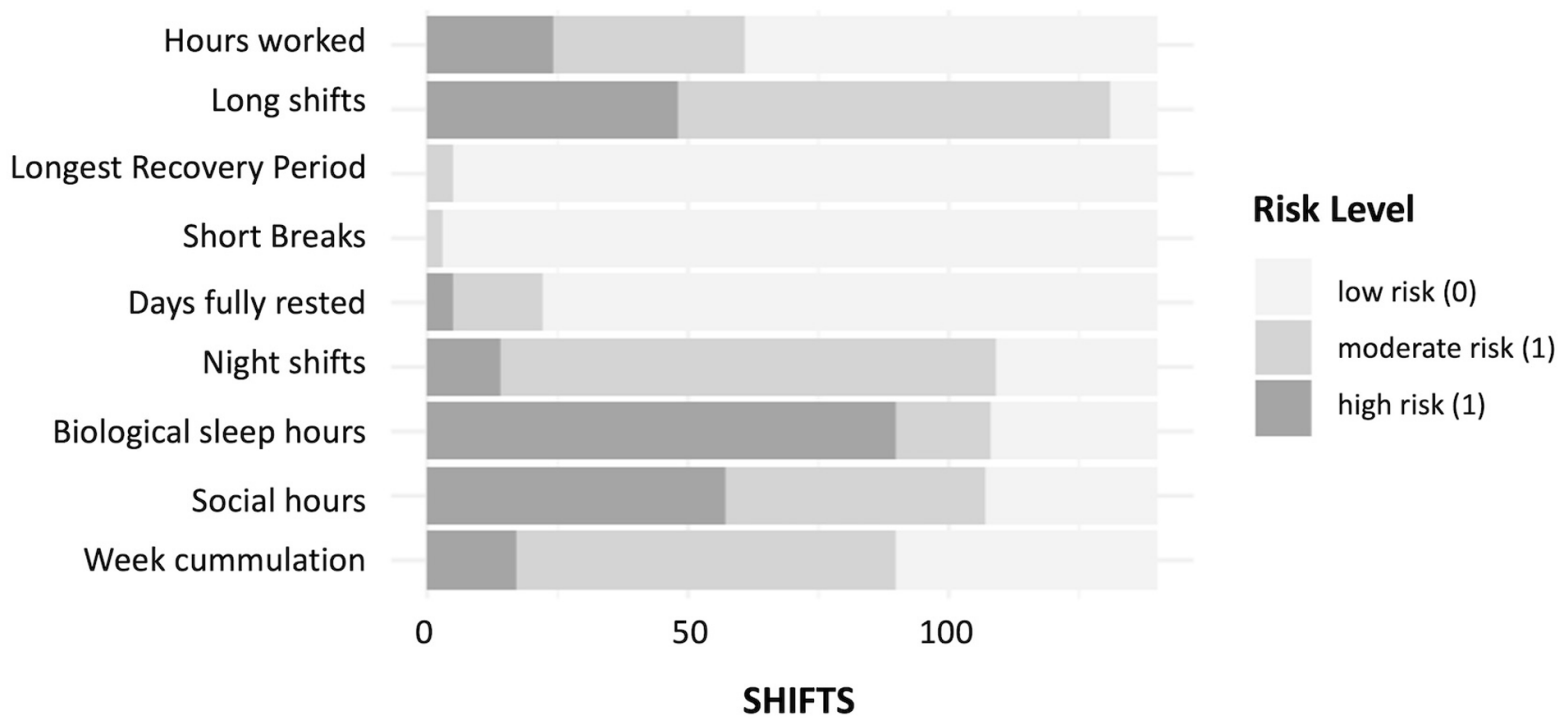
Distribution of risk levels across the nine items of the extended Shift Load Index, with bars representing the proportion of shifts at each risk category for each item.

In the theoretical testing phase, schedule analysis included 140 shifts worked by 55 EHPs (Figure 1, flow chart), with each participant providing repeated measures (two to three shifts each). Night shifts accounted for 51% of the shifts. The risk distribution across the extended Shift Load Index (SLI) with its 9 items is illustrated in Figure 2. While most SLI items exhibited varied risk levels, those related to rest opportunities generated minimal risk within this sample.

A total of 21% of shifts (*n* = 29) yielded SLI scores above 9, indicating elevated risk for insufficient recovery. The average FAID-FATIGUE score was 56.4 (± 24.3), with 19.5% of shifts demonstrating a peak score above the established threshold of 80. The average FAID-ALERTNESS score was 6.3 (± 1.8; corresponds to “some signs of sleepiness”), with 20.3% of shifts showing a peak KSS score of 8 or above (“sleepy, but some effort to keep awake”).

Given the variability of these outcomes across shift types, shift type was included as a random effect in the GLMM.

The GLMM showed that SLI was a significant predictor of both FAID-FATIGUE (*β* = 3.91, *p* < 0.001) and FAID-ALERTNESS (*β* = 0.02, *p* < 0.001), highlighting the impact of shift load on predicted fatigue and alertness levels. When SLI items were analyzed individually in separate models, the explanatory power increased substantially by adding shift type (*R*^2^ = 70 ➔ 88% for FAID-FATIGUE; *R*^2^ = 51 ➔ 99% for FAID-ALERTNESS). Detailed item-level findings are provided in the preprint (28).

### 3.2 SLI – behavioral validation

**Figure 3:**
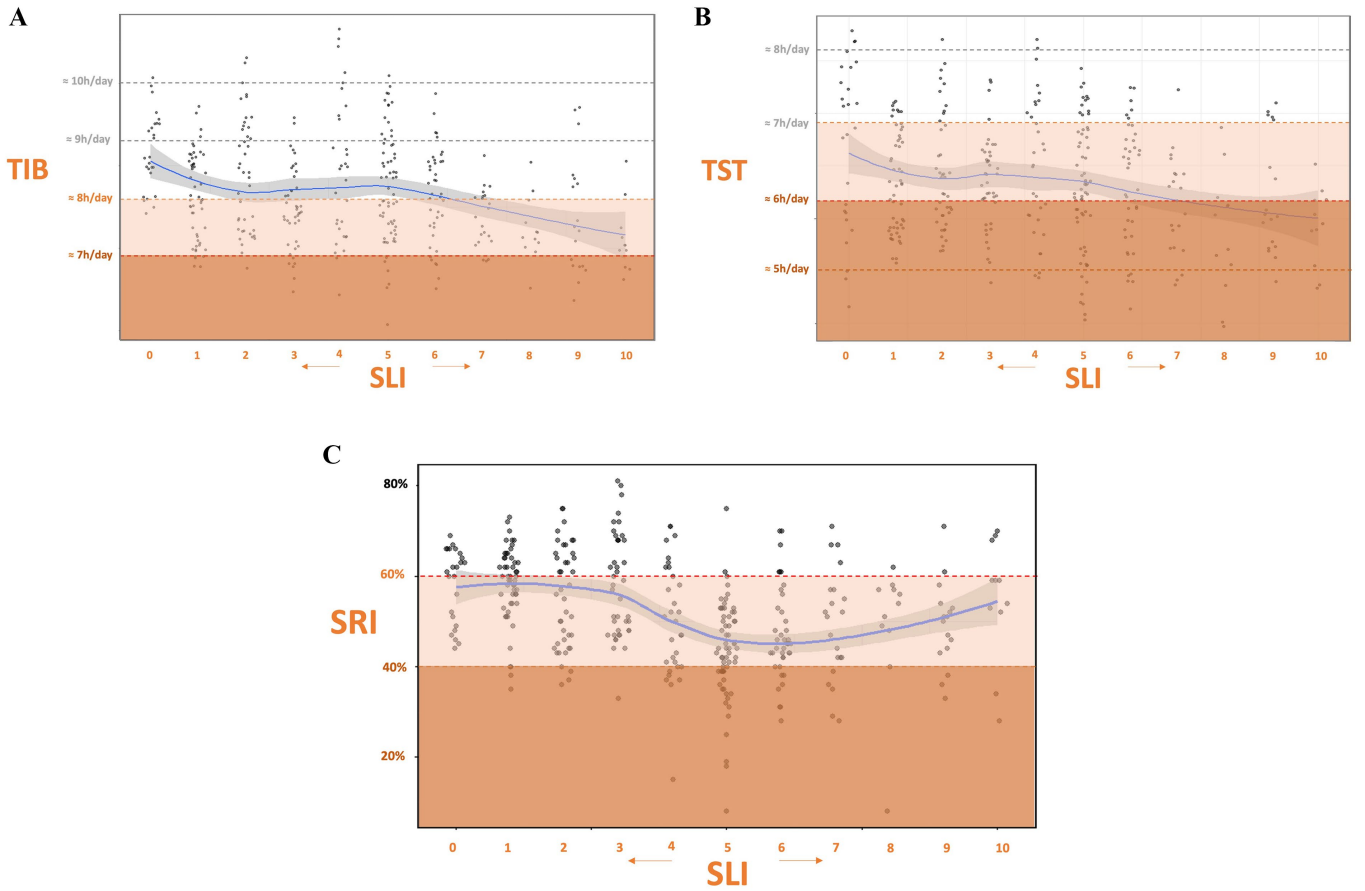
Associations between shift load and sleep measures. **(A)** Time in bed (TIB) versus Shift Load Index (SLI). **(B)** Total sleep time (TST) versus SLI. **(C)** Sleep Regularity Index (SRI) versus SLI. Blue lines show LOESS-smoothed trends. Generalized linear mixed models (controlling for subject) were all significant, indicating that higher SLI predicts reduced sleep quantity and regularity. Subjective zoning overlays guide visual interpretation.

The SLI in its eight item version was further assessed using objective sleep data from a subsample of 35 EHP (21 nurses, 14 physicians) each providing at least 7 consecutive 24 h recordings (totaling 360 observations; Figure 1). As shown in Table 2, participants SLI, TIB, TST, and SRI along with group comparisons by sex, profession, partner status, presence of young children, and chronotype are summarized. Descriptively, Figure 3 shows that TIB and TST declined with increasing SLI-leveling off at moderate loads before dropping further at high loads. SRI was stable at low SLI, dipped mid-range, then rebounded at high SLI, forming an inverted-U.

**Table 2:** Shift Load Index scores and objective sleep data.

Variable	Subgroup	*n %*	SLI (risk score)		TIB (hh:mm)			TST (hh:mm)			SRI (%)		
			*M*	SD	*M*	SD	*p*	*M*	SD	*p*	*M*	SD	*p*
All			4.06	2.67	8:07	0:55		6:12	0:50		51.9%	12.1%	
Sex	Male	34%	3.86	2.83	8:18	0:47		6:15	0:58		51.0%	10.4%	
	Female	66%	4.16	2.58	8:03	0:58	**	6:10	0:46	n.s.	52.4%	12.9%	n.s.
Profession	Physician	40%	4.21	2.94	7:48	0:44		6:08	0:48		56.5%	12.5%	
	Nurse	60%	3.96	2.46	8:20	0:59	***	6:15	0:52	n.s.	48.7%	10.8%	***
Site	ED 1	40%	4.09	2.11	8:21	1:00		6:41	0:44		51.0%	13.6%	
	ED 2	60%	4.05	2.92	7:59	0:52	***	5:57	0:46	***	52.4%	11.3%	n.s.
Status	Couple	57%	3.71	2.65	8:14	0:49		6:27	0:43		55.3%	11.3%	
	No couple	43%	4.57	2.62	7:57	1:03	*	5:50	0:52	***	47.2%	11.6%	***
Young Children	Yes	23%	4.06	2.44	8:05	0:58		6:16	0:42		54.4%	11.1%	
	No	77%	4.06	2.73	8:07	0:55	n.s.	6:10	0:52	n.s.	51.2%	12.3%	*
Chronotype	Morning	17%	4.08	2.80	7:41	0:43		5:58	0:39		54.3%	X8.5%	
	Neutral	71%	4.06	2.64	8:13	0:58		6:15	0:51		52.2%	12.9%	
	Evening	11%	4.06	2.61	8:21	0:41	***	6:15	1:04	*	44.0%	10.4%	*

SLI, Shift Load Index; TIB, Time in Bed; TST, Total Sleep Time; SRI, Sleep Regularity Index. All key variables were measured for a consecutive week, multiple weeks per subject were report in a sliding window approach. Weekly sleep volume was computed into a 24 h average. For dependent sleep variables group comparisons were performed with *t*-tests or ANOVA. (*n* = 360) *p < 0.05. **p < 0.01. ***p < 0.00.

**Figure 4:**
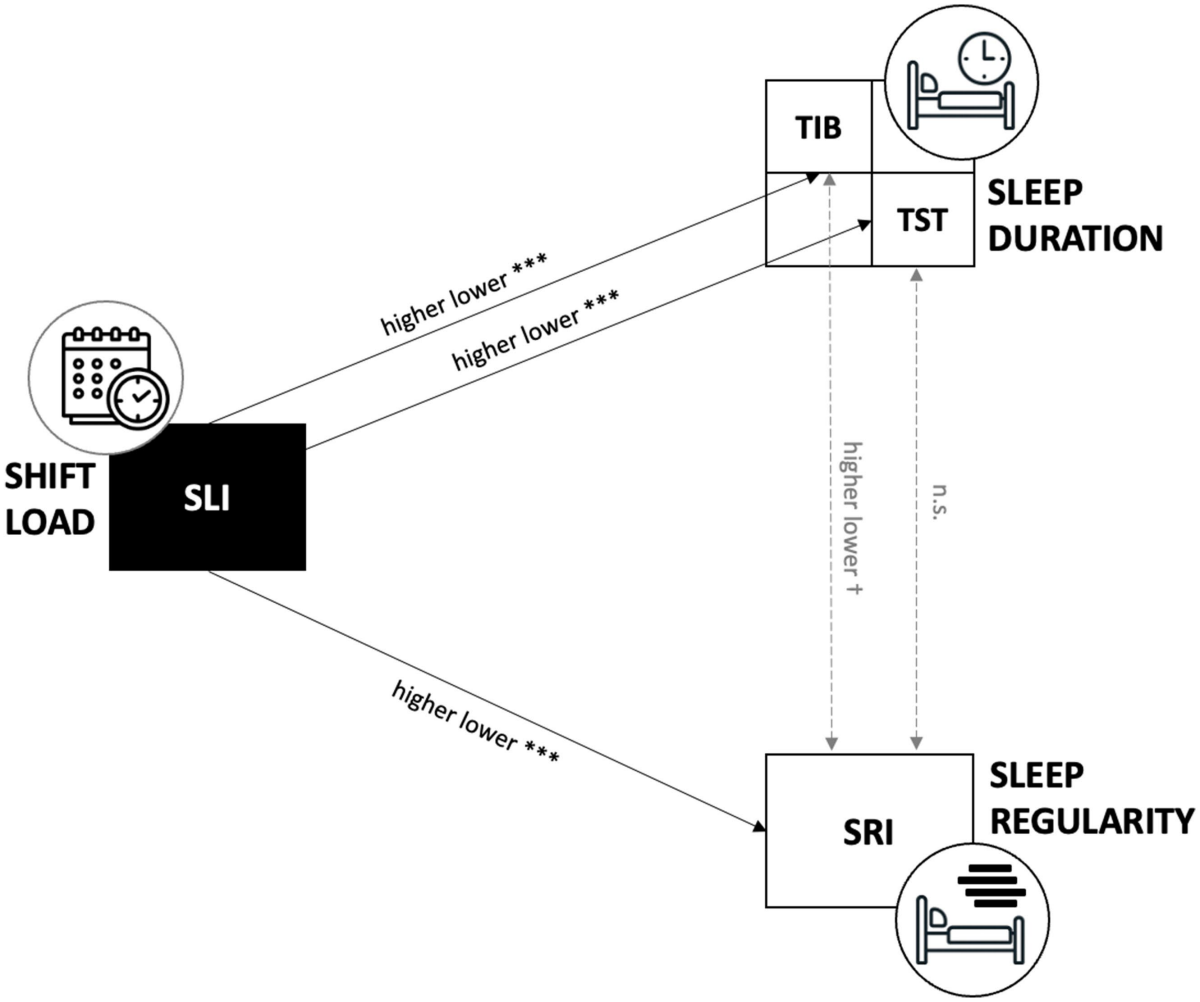
Conceptual model of shift-load effects on sleep. Schematic illustrating significant (solid arrows, *** *p* < 0.001) and non-significant (dashed arrow) pathways between SLI, TIB, TST, and SRI.

To assess the influence of shift scheduling on sleep outcomes, GLMMs were constructed with SLI as a predictor and subject as a random effect (Figure 4). SLI significantly predicted TIB and TST (respectively *β* = −0.53, *p* < 0.001, marginal *R*^2^ = 5% and *β* = −0.29, *p* < 0.001, marginal *R*^2^ = 2%). indicating that for each one-point increase in SLI, TIB decreased by approximately 32 min and TST by 17 min. SLI also significantly predicted lower sleep regularity (*β* = −1.06, *p* < 0.001), with a marginal *R*^2^ of 5%. With every increase of one point in the risk index, SRI decreased by approximately 1%. As a control, GLMMs with subject as a random effect showed that SRI exhibited only a non-significant trend with TIB (*β* = −0.24, *p* = 0.053; marginal *R*^2^ = 2%) and no association with TST (*β* = 0.02, *p* = 0.894), confirming that sleep regularity and sleep duration were not meaningfully associated.

The associations between shift load and individual sleep remained robust after adjusting for sex, age, BMI, weekly sport hours, young children, couple status, profession, shift experience, commute time and chronotype. In the adjusted models, the effect of SLI on TIB and on TST remained significant (respectively *β* = −33.19, *p* < 0.001, marginal *R*^2^ = 15% and *β* = −18.30, *p* < 0.001, marginal *R*^2^ = 20%), as did the effect of SLI on SRI (*β* = −1.06, *p* < 0.001; *R*^2^ of 21%). Further, none of the covariates independently predicted these outcomes.

### 3.3 Shift adaptation framework

**Figure 5:**
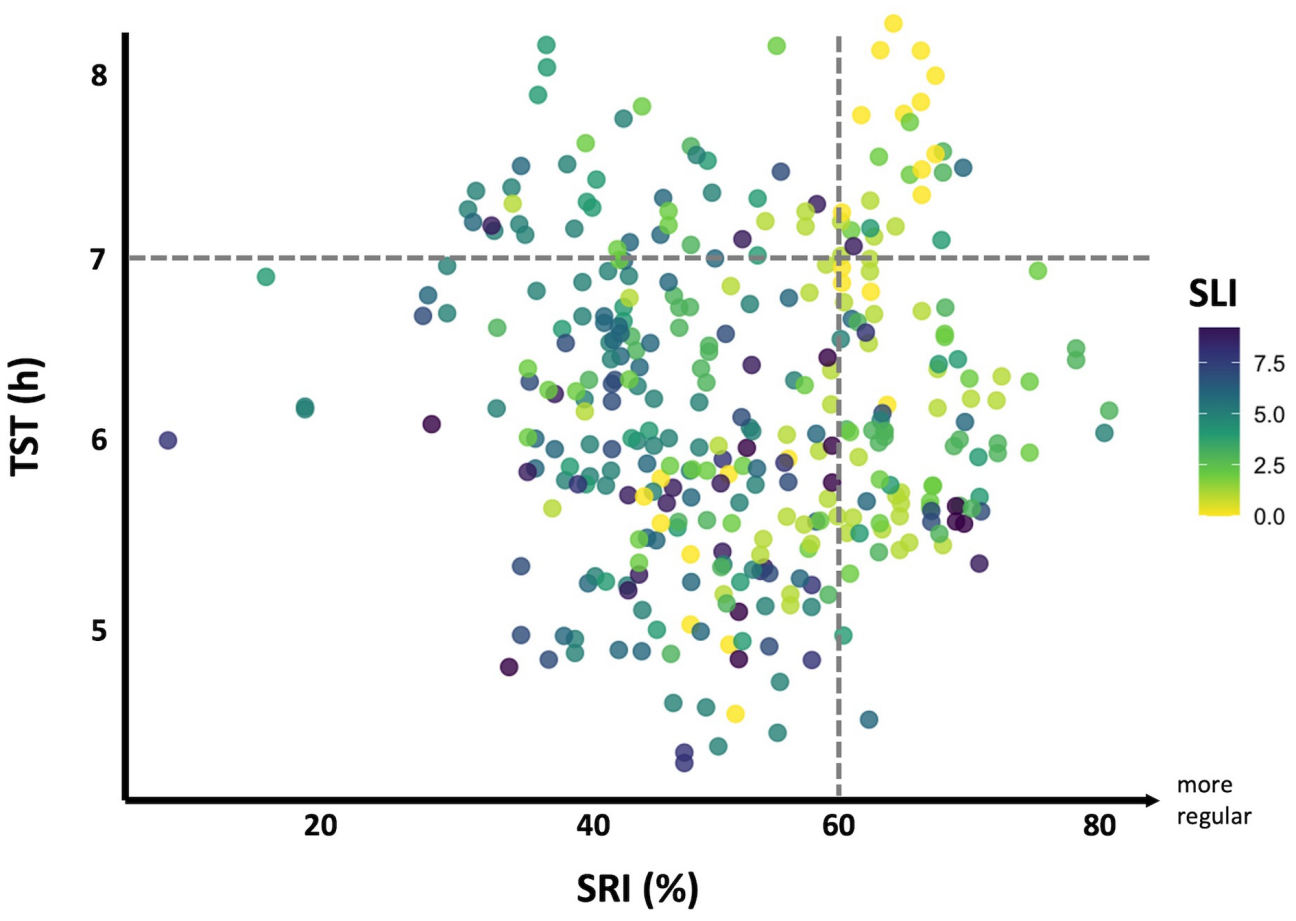
Shift adaptation framework. Scatterplot of SRI (%) versus average TST (h), with points colored by SLI on a Viridis scale (bright yellow = low shift load; dark purple-blue = high shift load). Dashed horizontal line at 7 h denotes the minimum recommended TST (65); dashed vertical line at 60% SRI marks the lowest quintile cutoff (61–64%) from non–shift-working cohorts (13, 30, 31).

Following the predictive value of SLI on behavioral sleep parameters, we plotted SRI against TST, with point color indicating the observed SLI (Figure 5). The exploratory multivariable regression of SLI on SRI and TST yielded a fitted prediction surface:


(2) SLI_pred=−0.0851SRI−0.0805TST+11.7760

GLMM revealed that SRI and TST were both significantly associated with SLI, yielding a total explanatory *R*^2^ of 48%, with fixed effects accounting for 18% of the variance. This interindividual variance in shift adaptation can be captured by calculating each individual’s residual (Observed SLI – Predicted SLI), we can identify those who fall outside the model’s confidence bounds (CI 95% [−0.516, 0.664]), highlighting participants who significantly outperform or underperform relative to their peers. For example, a positive residual signifies that a person handled a higher shift load than their sleep patterns would predict, indicating better adaptation to demanding schedules. Approximately 42% had residuals below −0.516, signifying worse adaptation and heightened sleep vulnerability.

## 4 Discussion

This study investigated the impact of shift load on sleep patterns among emergency healthcare professionals (EHPs), with a particular focus on the role of recovery limitations in predicting sleep duration and regularity. Higher Shift Load Index (SLI) scores, reflecting greater shift-related strain, were significantly associated with shorter sleep duration and reduced sleep regularity, even after accounting for chronotype and shift work experience. These findings underscore the substantial challenges EHPs face in preserving restorative sleep under demanding schedules, contributing to circadian rhythm misalignment and heightened risk of insomnia (35). In our study, EHPs spend approximatively 8 h in bed but effectively sleep for only 6 h underscores the widespread issue of chronic sleep debt among this population. These sleep disturbances have been associated with a spectrum of health issues, such as obesity (36) and an increased susceptibility to mental health disorders (37). Our results underline the critical need for effective management of work- rest cycles in healthcare environments to protect and promote both the well-being of professionals and the safety of patients.

### 4.1 Validation of the shift load index

The Shift Load Index (SLI) was developed by adapting a pre-existing risk assessment matrix to align with French work regulations, specifically targeting recovery opportunities within emergency department shift schedules. The SLI incorporates key factors such as working hours, shift length, night shifts, and the loss of biologically and socially meaningful time. In doing so, the present work extends the original Australasian scheme by calibrating it to European regulations and incorporating a social-rhythm component, thereby capturing aspects of circadian and social disruption not accounted for in earlier models. Interestingly, recovery periods contributed minimally to risk scores in this cohort, likely due to strict regulatory constraints. During the theoretical validation phase, the SLI demonstrated strong predictive validity for fatigue, aligning with results from two established biomathematical models: the Three Process Model of Alertness and the FAID model. While the former focuses on immediate sleep–wake patterns and daytime sleep, the latter accounts for cumulative fatigue over a week, integrating both physiological sleep pressure and social pressures. The SLI’s alignment with both models reinforces its ecological validity, particularly in identifying high-risk shifts for fatigue accumulation. Importantly, unlike the original scheme, which relied exclusively on self-reported fatigue and questionnaire data, our study addresses the missing field validation of this type of streamlined index against objective sleep metrics. Across models, every fifth shift in our cohort presented a heightened risk in terms of recovery and alertness. In comparison, a recent, larger study of British anesthesia residents reported only 12.7% of shifts exceeding elevated FAID fatigue thresholds, compared to our 19.5%, suggesting that French emergency department working hours may pose a higher burden (38). However, it should be noted that the Roche et al. study included residents and a much larger number of shifts, while our analysis focused only on full-time nurses and physicians, which may affect direct comparability.

Our findings indicate that maintaining adequate sleep becomes increasingly challenging as shift load intensifies, with SLI scores of 6 or above associated with significant sleep disruption. Given its robust associations with objective sleep metrics, SLI appears to be a reliable and practical rating system offering practical advantages for hospital management, risk assessment and application in real-world field settings.

### 4.2 Shift adaptation

Our proposed framework on shift adaptation lays the groundwork for a more nuanced understanding of the interplay between work schedules and individual differences in sleep response. While the SLI effectively captured sleep deficits associated with shift demands, our analysis revealed that approximately one-third of the variance in sleep adaptation was attributable to individual factors. This underscores the existence, within this challenge, of a modifiable margin for individual adaptation or vulnerability to shift schedules. Sleep behavior is highly individual and varies across schedules, suggesting that while work schedules have a tangible impact, adaptation can vary significantly depending on the risk profile, for instance morning type, age and family obligations intervene with shift adaptation (35). Recognizing this potential for adaptation supports the development of more personalized strategies for shift scheduling and fatigue management.

Building on this, the shift adaptation framework provides a conceptual basis for translating SLI outputs into actionable strategies. In practice, it could help identify vulnerable populations for targeted fatigue management, inform schedule design by highlighting high-risk sequences, and support occupational health monitoring at both the individual and organizational level. At present, the framework remains conceptual, and future studies should evaluate its value in diverse hospital systems while examining whether integrating SLI-based adaptations can reduce fatigue, improve sleep efficiency, and enhance clinical performance. The personalized shift adaptation framework seeks to pave the way for substantial improvements in clinical performance and organizational health.

### 4.3 Sleep regularity index

Sleep regularity is increasingly recognized as a critical determinant of physical and mental health (23, 24). It is closely modulated by lifestyle and environmental factors such as light exposure, food intake, and behavioral routines (39–41). In the context of emergency departments, exposure to artificial brightly light during night shifts may further disrupt circadian rhythms, complicating efforts to maintain consistent sleep regulation (42, 43). Although the SLI effectively captured overall sleep disruption, the specific analysis of sleep regularity revealed a U-shaped curve, largely driven by the frequency of night shifts, consistent with previous simulation studies (25). However, high SRI scores under high shift load may not necessarily reflect healthy sleep behavior, but rather stable, biologically inverted, and ultimately unhealthy patterns. Our findings are not surprising in that they confirm, both theoretically and in the field, that shift work produces irregular sleep patterns (25, 44). Critically, however, this is the first study in emergency healthcare professionals to quantify the extent of reduced sleep regularity with the SRI: the majority of participants fall below the 20% lowest-quintile cutoff established in non–shift-working populations. This stark shift relative to normal cohorts highlights the substantial health constraints imposed by these demanding schedules.

### 4.4 Clinical implications

Our findings suggest that sleep deficits among EHPs stem more from reduced sleep efficiency than insufficient time in bed, emphasizing the need for interventions that enhance both sleep quantity and quality (45, 46). Beyond work schedules, environmental factors are increasingly recognized as key determinants of sleep health. Variations in thermal comfort, noise, light exposure, and air pollution can compromise sleep initiation and maintenance and may differentially increase vulnerability, particularly in urban environments such as in our study sample (47, 48). Our observations align with Borbély’s two-process model of sleep regulation, which highlights the importance of addressing both sleep duration and regularity (49, 50). In high-stress environments like emergency departments, chronic sleep disruption may exacerbate compassion fatigue and burnout as well as general health, further underscoring the need for targeted sleep interventions (51, 52).

### 4.5 Organizational implications

The consequences of sleep deprivation extend beyond individual health, affecting both care quality and institutional costs. Fatigue-related medical errors alone cost health care systems billions annually (53), while high turnover rates in emergency services, driven by shift work and workplace stressors, cost an estimated 6,000 euros per departure (54). Institutional strategies must therefore move beyond mere scheduling adjustments to implement systemic workload monitoring, optimize staffing models, and foster a culture that proactively addressed fatigue risks (44, 55). Addressing critical incidents, workplace violence, and understaffing may further alleviate chronic stress and prevent burnout (56) as health problem (57).

### 4.6 Interventions to mitigate risk

Despite well-documented risks associated with shift work-related sleep disruption, evidence-based interventions remain underexplored. Our findings suggest that individual variability may outweigh shift schedule impacts, highlighting the need for targeted strategies on both organizational and individual levels (9, 58). Importantly, emerging evidence points to a genetic basis for individual vulnerability to sleep loss. Specific polymorphisms have been associated with differential responses to sleep deprivation in terms of cognitive performance, alertness, and subjective fatigue (59, 60).

At the organizational level, implementing programs that include sleep hygiene education, stress management training, and light exposure protocols can help mitigate sleep disruption and circadian misalignment (61). Additionally, tailored guidance on sleep practices, coping strategies, and shift-specific recovery techniques on an individual and chronotype level may significantly improve sleep quality and reduce fatigue, especially in high-stress settings like the emergency departments (62, 63). Orchestrated efforts have the potential to reduce sleep deprivation in healthcare settings.

### 4.7 Study limitations and future directions

While our study provides valuable insights, several limitations should be acknowledged. First, our data did not include cumulative shift burden from the previous week, limiting our ability to assess extended recovery deficits. Second, the behavioral validation was based on a modest sample drawn from two emergency departments within a single European city, limiting generalizability to other emergency departments or shift-working contexts. The modest sample size may also reduce statistical power, even though the repeated-measures design increases robustness. Third, the risk value distribution was skewed, generally reflecting lower-risk shifts, thereby constraining the analysis of those at higher risk levels. Fourth, our methodological choice to use a sliding window approach, while valuable for capturing time-series data, may have inadvertently introduced data redundancy.

Additionally, potential multicollinearity among SLI items raises the question of whether further simplification of the index may enhance utility without compromising predictive accuracy. Moreover, the current SLI does not account for excessive sleep, which may reflect compensatory behaviors and could carry its own risks. Lastly, our SRI metric did not account for sleepiness during activity intervals, overlooking diurnal sleepiness and nocturnal wakefulness patterns.

Future studies should address these gaps by incorporating more granular assessments of sleep–wake dynamics, particularly in diverse shift work settings. Expanding the dataset and applying the SLI in varied contexts will be crucial to validating and extending the framework’s applicability.

## 5 Conclusion

This study underscores the critical role of structured recovery management in mitigating sleep-related consequences of shift work among EHPs. By highlighting the predictive capacity of the SLI and exploring its associations with sleep quantity and regularity, we provide a practical framework for identifying high-risk shifts and vulnerable individuals. Specifically, we address three key gaps: the lack of objective validation of existing risk metrics, their limited calibration to European healthcare contexts, and the absence of SRI applications in shift workers. By bridging these gaps, our findings provide a stronger evidence base for integrating recovery-based indices into clinical scheduling and occupational health strategies. Integrating these findings into targeted intervention programs holds promise for reducing sleep disruption and enhancing both clinical performance and organizational health. Future research should explore the SLI’s integration into automated scheduling and fatigue monitoring systems, facilitating longitudinal analyses of shift adaptation and occupational well-being.

## Data Availability

The raw data supporting the conclusions of this article will be made available by the authors, without undue reservation.
